# Administration of erythropoietin exerts protective effects against glucocorticoid-induced osteonecrosis of the femoral head in rats

**DOI:** 10.3892/ijmm.2014.1644

**Published:** 2014-02-05

**Authors:** SEN CHEN, JIANPING LI, HAO PENG, JIANLIN ZHOU, HONGSONG FANG

**Affiliations:** 1 Department of Orthopedics, Renmin Hospital of Wuhan University, Wuhan, Hubei 430060, P.R. China; 2 Dongfeng General Hospital, Hubei University of Medicine, Shiyan, Hubei 442008, P.R. China

**Keywords:** erythropoietin, glucocorticoid, osteonecrosis, rat

## Abstract

Accumulating evidence has indicated that erythropoietin (EPO) plays a role in anti-apoptosis and tissue protection in a number of human diseases. The present study was implemented to evaluate these anti-apoptotic and tissue-protective effects in glucocorticoid-induced osteonecrosis in rats. Osteonecrosis was induced by low-dose lipopolysaccharide and subsequent high-dose methylprednisolone pulse. Rats in the preventive group were treated with 500 U/kg/day recombinant human EPO (rhuEPO) for 1 week. Hematological and histomorphometric methods were then used to determine the effects of the administration of rhuEPO. An analysis of trabecular bone architecture was performed to evaluate bone mass change in the osteonecrosis zone. Terminal deoxynucleotidyl transferase-mediated dUTP nick end-labeling (TUNEL) assay was performed to determine the apoptotic index of osteoblasts and osteocytes. Immunoblot analysis was performed to assess the expression of caspase-3 and vascular endothelial growth factor (VEGF) in the femoral head. Treatment with rhuEPO greatly improved the histological performance. Additionally, the incidence of osteonecrosis markedly decreased in the rats in the rhuEPO-treated group (22.2%) compared with the control group (66.7%). Furthermore, the expression of caspase-3 markedly decreased in the rhuEPO-treated group. Consistently, the apoptosis of osteoblasts and osteocytes, as determined by TUNEL assays, was inhibited following the administration of rhuEPO. By contrast, the expression of VEGF increased in the osteonecrosis zone in the rats treated with rhuEPO. The results from the present study demonstrate that EPO exerts prominent protective effects against glucocorticoid-induced osteonecrosis of the femoral head in rats by inhibiting the apoptosis of osteoblasts and osteocytes and increasing the expression of VEGF.

## Introduction

Osteonecrosis (ON) is a pathological process that predominantly impairs the femoral head and gradually progresses to the fracture of the subchondral bone, the collapse of the femoral head surface and the destruction of the hip joint. Although the etiology of ON has been attributed to a number of factors, glucocorticoid (GC) administration is the predisposing causative factor most commonly associated with the development of ON. Compromised vascularity and bone ischemia represent the traditional etiological background of ON. It was then realized that osteoblast and osteocyte apoptosis, but not compromised vascularity, is the primary etiology of GC-induced ON (1–12). In addition, the expression of vascular endothelial growth factor (VEGF), a key regulator that couples angiogenesis, bone formation and repair, reduces accompanied by the increase of osteoblasts and osteocytes apoptosis (13). Also, the compromised expression of VEGF has been found to impair the bone regeneration in the necrotic zones of the femoral head (14). On the basis of the above considerations, suppression of the apoptosis of the osteoblast and osteocytes and stimulation of the expression of VEGF could be two effective attempts in preventing the GC-induced ON of the femoral head.

Erythropoietin (EPO) is a pleiotropic cytokine originally identified for its role in erythropoiesis (15). Accumulating evidence has indicated that erythropoietin exerts anti-apoptotic and tissue-protective effects in a variety of human diseases, such as myocardial infraction (16–18), diabetes mellitus (19), spinal cord injury (20–22), ischemia-reperfusion (I/R)-induced kidney injury (23) and acute lung injury (24,25). Galeano *et al* reported that recombinant human EPO (rhuEPO) may be an effective therapeutic approach for improving clinical outcomes by enhancing the wound content of vascular endothelial growth factor (VEGF) following thermal injury (26). Rezaeian *et al* also demonstrated that the pharmacological manipulation of ischemic musculocutaneous tissue with 3 repetitive doses of EPO (500 IU/kg) upregulated inducible nitric oxide synthase (iNOS) and VEGF expression, and reduced apoptotic cell death and inflammation in the absence of any hematopoietic effect (27). Additionally, Holstein *et al* found that treatment with EPO upregulated the expression of VEGF during the early phase of bone defect healing, as shown by immunoblot and immunohistochemistry analyses (28). These data suggest that EPO exerts tissue protective effects through a VEGF-related pathway.

Therefore, we hypothesized that the administration of EPO can protect the femoral head from GC-induced ON by inhibiting apoptosis and increasing the expression of VEGF. We investigated this hypothesis using rats and a variety of methods, including histological staining and protein biochemistry. Indeed, we found that the administration of EPO markedly reduced the incidence of GC-induced ON in rats. Moreover, EPO suppressed the apoptosis of osteoblasts and osteocytes and increased the expression of VEGF.

## Materials and methods

### Animals

All experimental procedures adhered to the recommendations of the Experimental Animal Center of Wuhan University, Wuhan, China and the US Department of Health Guide for the Care and Use of Laboratory Animals, and were approved by the Ethics Committee of Wuhan University. A total of 54 male Wistar rats (10 weeks old) were obtained from the Hubei Provincial Center for Disease Control and Prevention, Wuhan, China. The rats were housed in a temperature- and humidity-controlled environment with unlimited access to food and water and a 12-h light/dark cycle.

### Experimental protocols

A total of 54 rats were divided equally into 2 groups: the control and EPO group. A rat model of ON was created by a sequential drug administration. The animals were administered 2 mg/kg lipopolysaccharide (LPS, from *Escherichia coli* 055: B5; Sigma, St. Louis, MO, USA) intravenously on days 0 and 1. On days 2, 3 and 4, the animals were administered 20 mg/kg methylprednisolone (MPS; Pfizer Pharmaceutical, Puurs, Belgium) intramuscularly. The animals in the EPO group were administered 500 U/kg rhuEPO (Shenyang Sunshine Pharmaceutical Co. Ltd., Shenyang, China) intramuscularly daily from day 0 for 7 days. The final day of administration was regarded as experimental week 0. The rats in the control group were not administered EPO. Nine rats in each group were sacrificed (6 for histological analysis and 3 for molecular biological analysis) on weeks 0, 2 and 4.

### Blood biochemistry

Blood was collected from the inferior vena cava at the time of sacrifice partially for regular testing and the remaining blood was centrifuged immediately. The supernatant was stored as platelet-rich plasma at −80°C. The triglyceride concentrations in the plasma were measured by using Triglyceride E-test kit (Nanjing Jiancheng Bioengineering Institute, Nanjing, China) according to the manufacturer’s instructions. The total cholesterol levels in the plasma were measured using the Cholesterol E-test kit (Nanjing Jiancheng Bioengineering Institute) according to the manufacturer’s instructions.

### Histopathological analysis

The proximal femurs were harvested and fixed with 10% formalin-0.1 M phosphate buffer, pH 7.4. After fixing in formalin, the bone samples were decalcified in 10% EDTA for 2 months. The decalcified bones were then embedded in paraffin and sectioned at 5 μm for histopathological analysis and immunohistochemistry. The sections were processed for routine haematoxylin and eosin staining to evaluate the ON lesions using well established criteria (29–32). The diagnosis of ON was performed in a blinded manner by 3 of the authors on the basis of the diffuse presence of empty lacunae or pyknotic nuclei of osteocytes in the bone trabeculae, accompanied by surrounding bone marrow cell necrosis. Rats that had at least 1 ON lesion in the areas examined were considered as ON^+^, while those with no ON lesions were considered as ON^−^. The incidence of ON was defined as the numbers of ON^+^ rats divided by the numbers of total rats in each group.

### Trabecular bone architecture

**Figure 1:**
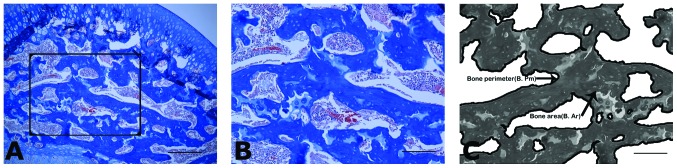
Trabecular bone microstructure in the femoral head. (A) The central region of the proximal epiphysis was selected for analysis. (B) The selected area was enlarged. (C) Digital image used for the quantification of trabecular architecture. Tissue area, trabecular bone area and trabecular bone perimeter were quantified and used to calculate trabecular bone architectural characteristics according to a parallel plate model. BV, bone volume; TV, tissue volume. Bone volume fraction (BV/TV) = B.Ar/T.Ar; trabecular thickness (Tb.Th) = [2/(B.Pm/B.Ar) ×1,000]; trabecular number (Tb.N) = (BV/TV)/Tb.Th; trabecular separation (Tb.Sp) = (1/Tb.N)-Tb.Th. Scale bar, (A) 400 μm; (B and C) 200 μm.

Frontal sections (4-μm-thick) of each femoral head were obtained and stained with Masson’s trichrome to highlight the microstructure. The trabecular tissue area (T.Ar, mm^2^), the trabecular bone area (B.Ar, mm^2^) and trabecular perimeter (B.Pm, mm) were quantified for each section in the central region of the proximal epiphysis; the bone volume fraction (%) was calculated as the trabecular bone area (BV) divided by the trabecular tissue area (TV) (Fig. 1). Other architectural properties, including trabecular thickness (Tb.Th, mm), trabecular number (Tb.N, mm^−^1) and trabecular separation (Tb.Sp, mm) were calculated according to a parallel plate model (33,34).

### TUNEL assays

Apoptotic osteoblasts and osteocytes were detected using the terminal deoxynucleotidyl transferase-mediated dUTP nick end-labeling (TUNEL) assay, with an In Situ Cell Death Detection Kit (Roche Diagnostics, Mannheim, Germany), according to the manufacturer’s instructions. Briefly, following routine deparaffinization and treatment with H_2_O_2_ (3%), the sections were digested with proteinase K (20 μg/ml, pH 7.4, 12 min) at 25°C and incubated with the reaction mixture (1:40, 60 min) at 37°C. Incorporated fluorescein was detected with horseradish peroxidase following incubation for 30 min at 37°C and were subsequently dyed with 3,3′-diaminobenzidine (DAB). Brown nuclei were assessed as positive apoptotic cells. The apoptotic index (AI) was evaluated for 1 section of 5 randomly selected high-power fields.

### Immunoblot analysis

Femoral heads dissected from the proximal one-third of the femur neck were powdered in liquid nitrogen by hand milling, followed by homogenization on ice-cold radioimmunoprecipitation (RIPA, Beyotime Institute of Biotechnology, Beijing, China) buffer containing phenylmethylsulfonyl fluoride (PMSF, Beyotime Institute of Biotechnology) and a cocktail of protease inhibitors (Complete, EDTA-free; Roche Diagnostics). Following sonication, the samples were centrifuged twice at 14000 rpm at 4°C for 10 min to remove cell debris, nuclei and large particulates. The supernatant containing the cytosolic protein fraction was then collected. A quarter volume of 5× loading buffer was added and boiled at 95°C for 5 min then stored at −20°C until electrophoresis. Proteins were separated by 10% sodium dodecyl sulfate polyacrylamide gel electrophoresis and transferred onto polyvinylidene difluoride membranes (Millipore Corp., Bedford, MA, USA). After being blocked with 2% bovine serum albumin (Roche Diagnostics), the membranes were incubated at 4°C overnight with rabbit anti-caspase-3 or anti-VEGF antibody (1:500, Santa Cruz Biotechnology, Santa Cruz, CA, USA) or rabbit anti-GAPDH antibody (1:500, Santa Cruz Biotechnology) as primary antibodies, followed by incubation with peroxidase-conjugated secondary antibodies (1:2000, Jackson Laboratories, West Grove, PA, USA) at 25°C for 1 h. The proteins on the membranes were visualized using an ECL plus detection kit (Amersham Pharmacia Biotech, Buckinghamshire, UK), exposed to Kodak X-ray film and processed using a scanner. The optical density of the bands was analyzed using Image-Pro Plus 6.0 software. The expression of caspase-3 and VEGF was then normalized to GAPDH.

### Statistical analysis

Data are presented as the means ± standard error of the mean (SEM). Statistical analysis was performed using SPSS 13.0 software (SPSS Inc., Chicago, IL, USA). One-way analysis of variance (ANOVA) with Turkey’s post hoc test was used to examine differences between groups. Statistical differences were considered significant when the P-value was <0.05.

## Results

### EPO administration does not affect body weight, red blood cell (RBC) count, hemoglobin and hematocrit levels in rats

**Figure 2:**
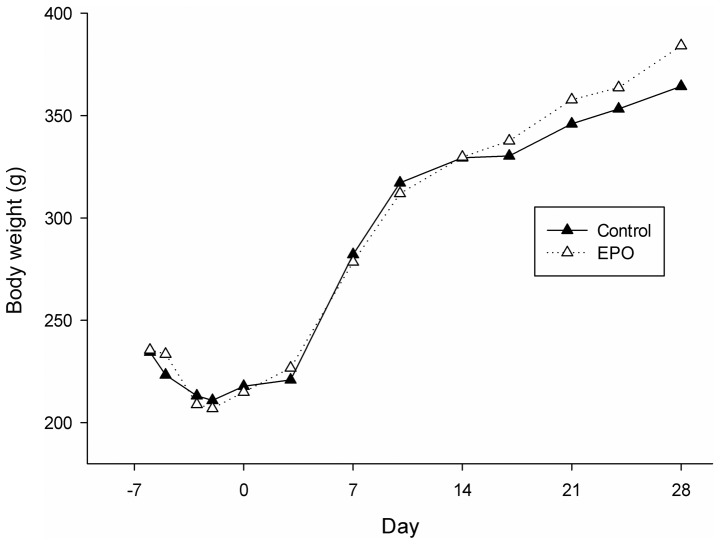
Body weight over time of rats in the control and erythropoietin (EPO) groups.

**Figure 3:**
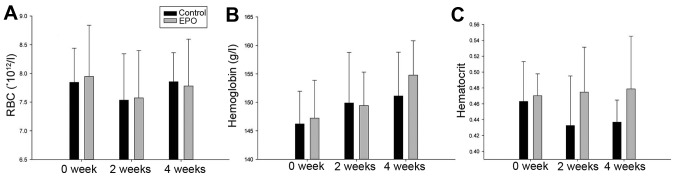
Blood tests over time in the control and erythropoietin (EPO) groups. (A) Red blood cell (RBC) count, (B) hemoglobin, (C) hematocrit. Results are presented as the means ± SD.

No accidental deaths took place during the experiment. The body weight of the animals in both groups decreased slightly during the 1st week (week, −1), and thereafter increased from weeks 0 to 4. There were no significant differences between the body weight of the animals in the 2 groups during the whole experimental period (Fig. 2). Blood analyses did not reveal a significantly higher RBC count in the rhuEPO-treated animals when compared with the controls. Additionally, the levels of hemoglobin and hematocrit displayed a similar trend (Fig. 3), as the statistical analysis revealed no significant differences between the groups. These data indicate that the appropriate dose of EPO does not affect body weight and blood components in rats.

### EPO administration decreases the plasma concentration of triglycerides and total cholesterol in rats

**Figure 4:**
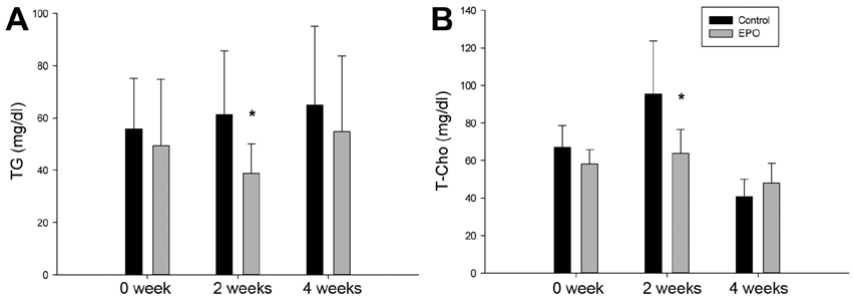
Blood biochemistry tests over time in the control and erythropoietin (EPO) groups. (A) Plasma levels of triglycerides (TG). (B) Level of total cholesterol (T-Cho) in the plasma. Results are presented as the means ± SD. ^*^P<0.05, compared to the control.

We then examined the concentrations of triglycerides and total cholesterol in plsma. As shown in Fig. 4, the plasma levels of triglycerides in the EPO group were lower than those in the control group. Notably, a significant difference was observed on week 2 (P<0.05). The level of total cholesterol in the plasma in the EPO group was also significantly lower than that in the control group on week 2. These results suggest that the appropriate dosage of EPO reduces the plasma concentration of triglycerides and total cholesterol during the early stages of ON.

### EPO administration improves histological performance and reduces the incidence of ON in rats

**Figure 5:**
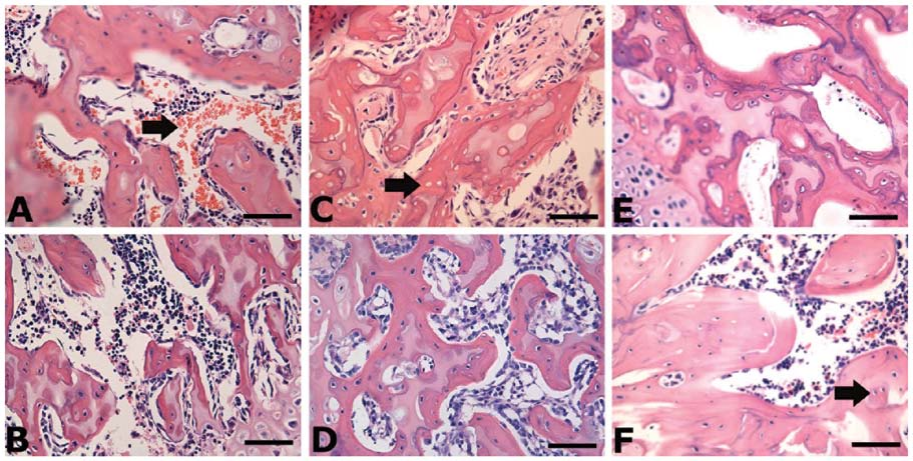
Histological changes of trabecular bone of femoral head. (A, C and E) Histological performance in the control group over time; (B, D and F) histological performance in the EPO group. Histological performance in the EPO group was much better than that in the control group. (A and B) On week 0, the necrotic bone trabeculae showed pyknotic nuclei of osteocytes and empty lacunae accompanied with the hemorrhage and necrosis of bone marrow. (C and D) On week 2, the necrotic bone trabeculae also showed pyknotic nuclei and empty lacunae, while the numbers of empty lacunae increased significantly. (E and F) On week 4, the necrotic bone trabeculae again showed much more empty lacunae and the appearance of scar tissue in the medullary space. The arrow in (A) indicates the hemorrhage and necrosis of bone marrow. The arrows in (C and F) indicate empty lacunae and pyknotic nuclei of osteocytes. Scale bar, 100 μm (A–F).

The presence of diffuse and empty lacunae or pyknotic nuclei of osteocytes in the bone trabeculae, accompanied by surrounding bone marrow cell necrosis, is defined as ON. To assess the ON lesions in the control and EPO groups, we performed haematoxylin and eosin staining to determine the histological characteristics (Fig. 5). On week 0, the necrotic bone trabeculae showed pyknotic nuclei of osteocytes and empty lacunae accompanied by the hemorrhage and necrosis of bone marrow (Fig. 5A and B). On week 2, the necrotic bone trabeculae also showed pyknotic nuclei and empty lacunae, while the numbers of empty lacunae markedly increased (Fig. 5C and D). Additionally, the fibrous tissue was found to accumulate in the medullary space (Fig. 5C and D). On week 4, the necrotic bone trabeculae showed further empty lacunae and the presence of scar tissue in the medullary space (Fig. 5E and F). Based on the histopathological characteristics, we determined the incidence of ON as presented in Table I. In the control group, the incidence of ON increased with time, whereas the administration of EPO prevented the occurrence of ON. The total incidence of ON markedly decreased in the EPO group compared with the control group (22.2 vs. 66.7%) (Table I), suggesting that the appropriate dose of rhuEPO can greatly reduce the incidence of steroid-associated ON in rats.

### Trabecular bone volume fraction and trabecular thickness increases upon EPO administration

**Figure 6:**
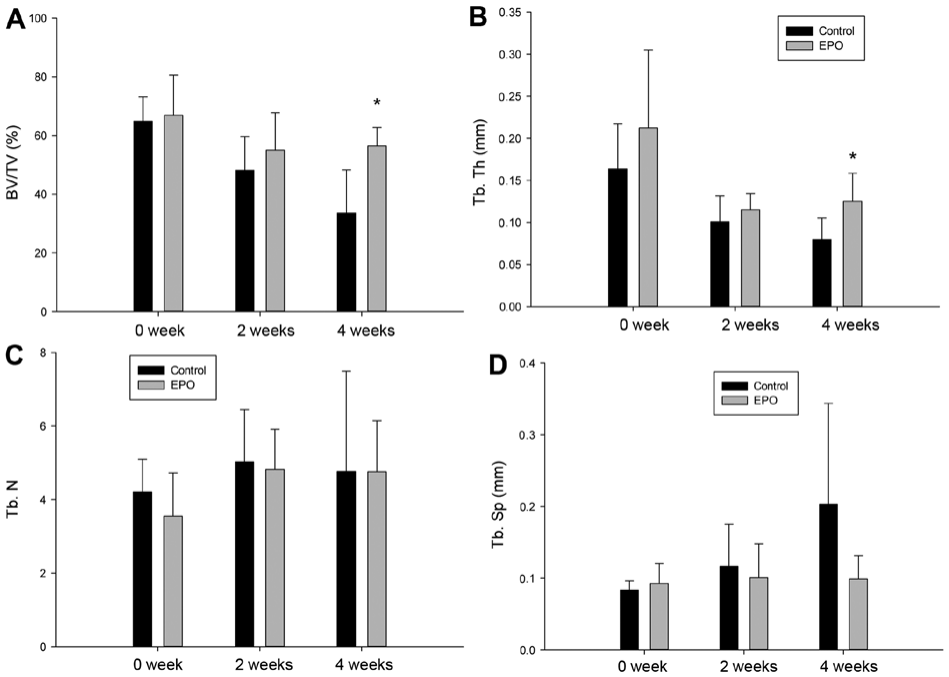
Trabecular bone microstructural properties. (A) Bone volume fraction (BV/TV) was significantly higher in the erythropoietin (EPO) group compared with the control group on week 4 (^*^P=0.006), but not at other time points. (B) Bone trabecular thickness (Tb.Th) was significantly higher in the EPO group compared with the control group on week 4 (^*^P=0.023). (C) There was no significant difference in trabecular number (Tb.N) between the 2 groups at the 3 time points. (D) There was no significant difference in trabecular separation (Tb.Sp) between the 2 groups at the 3 time points.

To assess the microstructural architecture of the trabecular bone, we introduced 4 indicators, including bone volume fraction, trabecular thickness, trabecular number and trabecular separation, as described in Materials and methods. No significant difference was observed as regards the 4 indicators between the 2 groups on weeks 0 and 2. Of note, the trabecular bone volume fraction and trabecular thickness in the EPO group increased with statistical significance on week 4 (P=0.006 and P=0.023; Fig. 6A and B). However, there were no significant differences observed in trabecular number and trabecular separation between the 2 groups (Fig. 6C and D).

### EPO administration inhibits apoptosis in the ON zone

**Figure 7:**
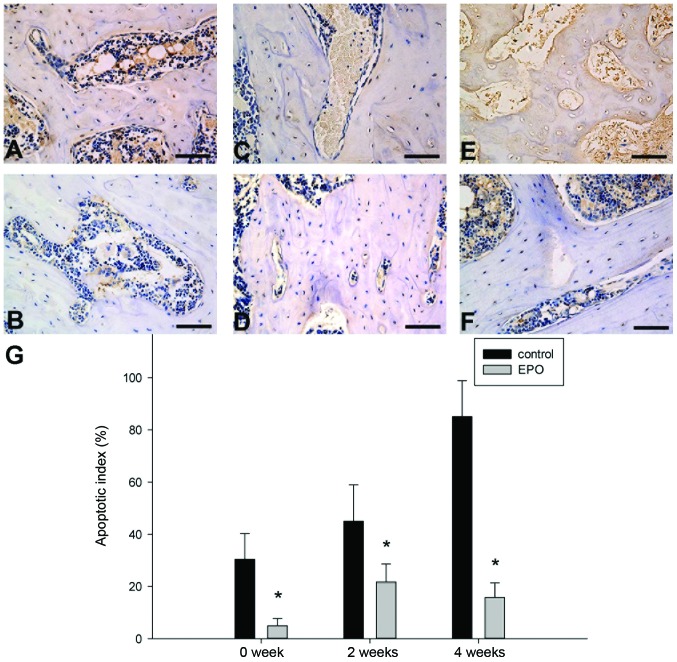
Photomicrograph of TUNEL staining showing evidence of apoptotic cells in the necrotic zone. (A, C and E) show the TUNEL-positive cells in the control group over time. (B, D and F) show the TUNEL-positive cells in the erythropoietin (EPO) group over time. Scale bar, 100 μm (A–F). (G) Quantitative analysis of TUNEL-positive staining in (A–F). Data are presented as the means ± SD. ^*^P<0.05 vs. controls.

To determine whether the administration of EPO affects apoptosis in the trabecular bone of the femoral head, we conducted TUNEL assays and found that positively stained cells were detected in the ON zones in both groups, although the number of TUNEL-positive cells varied at different time points (Fig. 7A–F). Additionally, treatment with EPO resulted in less apoptotic cells/high-power field, which was further confirmed by quantitative analysis showing a significantly lower apoptotic index in the EPO group compared with the control group at all the 3 time points (P<0.05; Fig. 7G). These data indicate that the administration of EPO protects the trabecular bone cells from apoptosis.

### EPO administration reduces caspase-3 and increases VEGF levels

**Figure 8:**
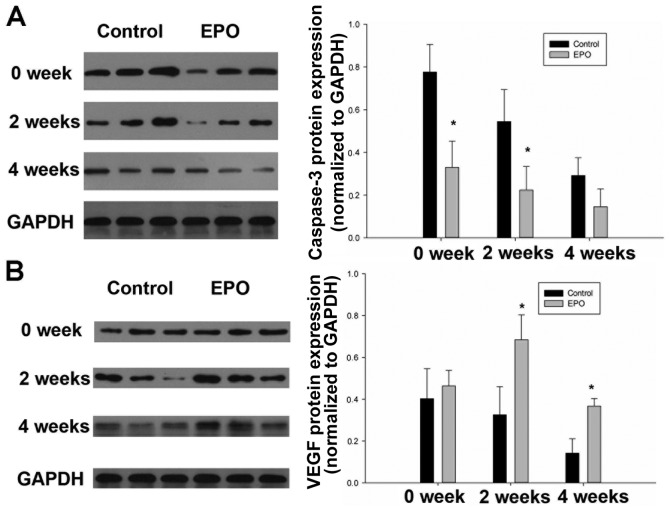
Immunoblot analyses of the expression of (A) caspase-3 and (B) VEGF in erythropoietin (EPO)-treated animals and the untreated controls at 0, 2 and 10 weeks. Data are presented as the means ± SD. ^*^P<0.05 vs. controls.

To further consolidate our previous observations, we performed immunoblot analysis to assess the expressions of caspase-3, an indicator of apoptosis, as well as the levels of VEGF. Indeed, the expression of caspase-3 within the femoral head markedly decreased in response to EPO treatment (Fig. 8A), while the difference was significant on week 0 and week 2 (P<0.05). On the contrary, the expression level of VEGF markedly increased following treatment with EPO on weeks 2 and 4 (Fig. 8B). These data demonstrate that EPO exerts tissue-protective effects against ON through 2 mechanisms, the inhibition of apoptosis and the enhancement of VEGF expression.

## Discussion

EPO has been found to initiate tissue-protective effects, including the prevention of apoptotic cell death (35) and the induction of angiogenesis and tissue regeneration (36), in addition to its regulatory function in erythropoiesis (37). These effects may be beneficial to the pathological process of ON. However, the excessive use of EPO can boost the hematocrit, which is detrimental for microcirculatory perfusion in ischemic situations, such as ON, and which causes polycythemia, a condition with abnormally high levels of RBCs. Rezaeian *et al* (27) reported that the administration of 5 repeated doses of 500 units of EPO only marginally increased the hematocrit in C57BL/6 mice after a period of approximately 7 days, whereas the administration of 5,000 units 5 times significantly increased the hematocrit. It has been found that a significantly increased hematocrit can aggravate bone necrosis due to impaired rheology, i.e., decreased RBC velocity and nutritive perfusion and hyperviscosity, which is believed to be detrimental despite the anti-apoptotic and angiogenic effects mediated by EPO (27). Furthermore, a previous study demonstrated that, to prevent the detrimental effects of EPO, the treatment of anemia in chronic renal failure usually requires low doses of EPO between 350 and 400 units or less on a very repetitive base over several weeks or months (27). Therefore, we decided to administer 7 doses of (500 units/kg/day) of EPO in our study. Blood analyses did not reveal a significantly higher RBC count in the EPO-treated animals compared with the untreated controls on weeks 0, 2 and 4 (Fig. 3). The levels of hemoglobin and hematocrit showed a similar trend (Fig. 3). Additionally, histological analysis revealed a lower ON index in the EPO group compared iwth the control group (Figs. 5 and 6). These results illustrate that the administration of EPO at the appropriate dosage and repetitive manner exerts beneficial effects rather than harmful effects on the pathological process of ON. In accordance with the former data, the apoptotic index of osteoblasts and osteocytes in the EPO group was greatly reduced compared with the controls (Fig. 7). Moreover, the expression of VEGF markeldy increased following treatment with EPO (Fig. 8B). In conclusion, the results from our study suggest that the tissue-protective function of EPO in GC-induced ON is possibly attributed to the combination of its anti-apoptotic and VEGF-enhancing effects.

GCs have been reported to elicit the apoptosis of osteoblasts and osteocytes through direct and indirect mechanisms. Osteoblasts and osteocytes can be the direct targets of GC action *in vivo* and excess levels of steroid hormones directly induce the apoptosis of these cell types (5). Alternatively, infarction, as well as oxygen and nutrient deprivation caused by a high-dose administration of GCs inevitably lead to the apoptosis of osteoblasts and osteocytes (7). The activation of caspase-3, a common downstream effector of apoptotic signaling pathways, including the involvement of direct and indirect mechanisms, is observed in the GC-induced apoptosis of osteoblasts and osteocytes (38,39). Consistent with the data from other studies (40,41), we also demonstrated that GC enhanced apoptosis, as well as caspase-3 expression in trabecular bone in a time-dependent manner (Figs. 7 and 8A). However, the elevated levels of apoptosis and caspase-3 expression were significantly repressed in response to EPO administration (Figs. 7 and 8A). However, it remains to be determined whether caspase-3 expression is causative to the apoptosis observed in the ON zone and whether caspase-3 is a direct target of EPO. Our results demonstrate that the appropriate dose of EPO exerts anti-apoptotic effects on GC-induced ON.

A number of of studies have suggested that GC impairs angiogenesis and osteogenesis by suppressing the production of VEGF in the femoral head in GC-induced ON (13,42–46). It has been reported that GCs suppress angiogenesis in fetal metatarsals, as well as hypoxia-inducible factor-1α transcription and VEGF production in osteoblasts and osteocytes (13). Osteoblasts derived from femoral heads have been shown to exhibit a decrease in VEGF expression within 24 h of incubation with GCs (43). Furthermore, it is likely that VEGF and its receptors play vital roles coupling osteogenic and angiogenic processes in adult bone during remodeling and repair processes (47). In an *in vivo* model of ON, rabbits treated with depomedrol plus adrenocorticotropic hormone (ACTH)for 1 month displayed fewer signs of trabecular necrosis and an increased expression of VEGF in comparison to animals treated with the steroid, depomedrol, alone (48). In the present study, immunoblot analysis revealed an elevated VEGF expression in the ON zone in the group administered EPO (Fig. 8B). Moreover, a higher VEGF expression was usually accompanied with a better histological performance and a higher trabecular bone volume fraction of the femoral head (Figs. 5 and 6). Taken together, the data from our study suggest that the administration of EPO exerts angiogenic and osteogenic effects in ON through a VEGF-related mechanism.

In conclusion, the present study demonstrates that the administration of EPO exerts prominent protective effects against GC-induced ON of the femoral head in rats by inhibiting the apoptosis of osteoblasts and osteocytes and increasing the expression of VEGF. Further investigations of the molecular basis of EPO-mediated anti-apoptosis and regeneration in ON are required in order to develop novel strategies for the prevention and treatment of GC-induced ON.

## Figures and Tables

**Table I:** Incidence of osteonecrosis (%).

Group	Week 0	Week 2	Week 4	Total
Control	33.3 (2)	66.7 (4)	100.0 (6)	66.7
Erythropoietin	16.7 (1)	33.3 (2)	16.7 (1)	22.2
χ^2^ test	0.4444	1.3333	8.5714	7.2000
P-value	0.5050	0.2482	0.0034	0.0073
